# Nicotine-free electronic vape fluid stimulates angiogenic processes *in vitro* through ARF6-mediated oxidative stress

**DOI:** 10.3389/ftox.2025.1699112

**Published:** 2026-01-22

**Authors:** Lewis Spurrier-Best, David Butcher, Evangelene Blackham-Hayward, Zsuzsanna Kertesz, Havovi Chichger

**Affiliations:** Biomedical Research Group, Anglia Ruskin University, Cambridge, United Kingdom

**Keywords:** electronic cigarette, endothelial cells, angiogenesis, neovascularisation, vascular endothelial growth factor, vascular biology

## Abstract

**Introduction:**

The increase in e-cigarette use in the population has led to substantial interest in the health impacts associated with e-cigarette smoking. E-cigarette smoking represents a key external environmental cell stressor. Whilst there have been several studies to investigate the effect of nicotine-containing e-cigarette fluid, there is still a significant lack of understanding of how nicotine-free e-cigarette smoking can impact individuals. However, preliminary studies indicate that nicotine-free e-cigarette smoking can cause impaired endothelial function in humans.

**Materials and Methods:**

In the present study, we therefore used a common brand of nicotine-free e-cigarette and human umbilical vein endothelial cells to assess angiogenic processes *in vitro*.

**Results:**

We observed a significant upregulation in endothelial cell adhesion, migration and new tube formation with exposure to nicotine-free e-cigarette condensate (eVape) which was abrogated with exposure to the antioxidant, N-acetyl cysteine. Proteome analysis demonstrated that eVape exposure increased expression of the pro-angiogenic factors, angiogpoeitin-2, endoglin (CD105), PIGF and VEGF, as well as the ADP ribosylation factor, ARF6, and ARF6-GEF, ARNO. Chemical inhibition of ARNO reduced eVape-induced oxidative stress, angiogenic processes, and release of angiogpoeitin-2, endoglin (CD105) and VEGF.

**Discussion:**

These findings demonstrate that nicotine-free eVape causes aberrant upregulated angiogenesis in an in vitro model of the human endothelium through ARNO-dependent signalling. This study is the first to demonstrate the molecular mechanisms in response to the cellular stressor, nicotine-free eVape which underlie impaired vascular function.

## Introduction

Electronic cigarettes (e-cigarettes) were commercially introduced to the market in 2007 as tools for cigarette-smoking cessation (Hartmann-Boyce et al., 2021). Whilst e-cigarettes provide the sensation of smoking a cigarette, they do not contain tobacco and are therefore deemed a safe method of smoking cessation (Barbeau et al., 2013). In adult populations, e-cigarettes are predominantly used by current or former smokers; however, in young people, there has been an alarming increase in new smokers using e-cigarettes (Du et al., 2020; Dai and Leventhal, 2019). In 2018, it was reported that approximately 15% of people aged 18–24 years in the USA used e-cigarettes compared to 9.2% in 2016 (Hammond et al., 2023). In a 2022 study conducted in the UK, this trend was even more stark with 24% of people aged 16–19 years reporting regular or occasional e-cigarette use (Chaumont et al., 2018a). Although we do not fully understand the long-term health impacts of e-cigarette use, there are many recent studies evidencing significant health concerns. For example, acute adverse pulmonary effects like airway flow resistance, oxidative stress, and inflammation have been routinely noted with the use of e-cigarettes (Ghosh et al., 2019; Keith and Bhatnagar, 2021; Xie et al., 2022). Analysis of the Population Assessment of Tobacco and Health (PATH) study data in 18–24 year olds shows significant increases in wheezing-related respiratory symptoms (Tilles, 2006); these symptoms typically precede a lengthy subclinical phase followed by clinical presentation of chronic respiratory conditions like asthma and chronic obstructive pulmonary disease (Gentry and Gentry, 2017; Alexander et al., 2020). Thus, there is a need to fully understand the health impacts of e-cigarette smoking with the aim of informing policy and education for young people as well as reducing potential health epidemics in the future.

One of the key clinical outcomes of e-cigarette smoking for which there are significant data is e-cigarette-associated lung injury (eVALI); this is a unique disease entity associated with rapid escalation in e-cigarette smoker incidence (Chatterjee et al., 2019). The molecular mechanisms underlying eVALI are linked to a pro-inflammatory switch in the pulmonary microvasculature leading to pulmonary oedema (Mohammadi et al., 2022; Schweitzer et al., 2015). Indeed, researchers have previously demonstrated the negative impacts of e-cigarette fluid on the lung endothelium along with breakdown of the endothelial barrier, excessive inflammation, and vascular leakage (Blackham-Hayward et al., 2024; Caporale et al., 2019). Whilst extant research mainly focuses on the pulmonary vasculature, there are some studies that implicate a wider range of vascular responses to e-cigarettes. Several studies have demonstrated elevated arterial stiffness and oxidative stress in the vasculature in individuals using e-cigarettes (Ghosh et al., 2019; Carnevale et al., 2016; Chaumont et al., 2018b; Franzen et al., 2018; Vlachopoulos et al., 2016; Michaud et al., 2006). These clinical findings suggest a direct role of e-cigarette use in the development of endothelial dysfunction throughout the vasculature.

In individuals who smoke cigarettes, it is well-understood that excessive vascular remodelling and impaired angiogenesis occur outside the lung through a nicotine-dependent pathway (Zhang et al., 2001a; Yingst et al., 2019). As e-cigarettes have been found to cause similar increases in the blood nicotine levels as cigarette smoking (Hajek et al., 2020; Ashour et al., 2020), it is not surprising that studies on e-cigarettes also report aberrant angiogenic signals. Interestingly, however, both excessive and abrogated angiogenesis induced by vascular endothelial growth factor (VEGF) have been observed for exposure to e-cigarettes depending on the model studied and e-cigarette type used (Chhor et al., 2023; Jaleel et al., 2021; Liu et al., 2022; Shi et al., 2019; Cucina et al., 2000a, 2000b). All these studies used e-cigarettes containing nicotine, a potent angiogenic stimulus acting through nicotinic-receptor-induced endothelial nitric oxide synthase (eNOS) signalling (Yingst et al., 2019; Heeschen et al., 2001; Mannell et al., 2012). However, we previously showed that nicotine-free e-cigarette condensate (eVape) also regulates eNOS signalling (Caporale et al., 2019); we demonstrated that eVape increases oxidative stress, inflammatory cytokine release, and vascular permeability in the lung endothelium (Caporale et al., 2019). These changes are supported by findings of reduced blood flow and impaired endothelial functions in individuals who smoked nicotine-free e-cigarettes (Carnevale et al., 2016). It is therefore likely that nicotine is not the sole factor causing vascular complications in e-cigarette smokers. In the present study, we investigated the effects of nicotine-free eVape on angiogenesis and the molecular mechanisms underlying new vessel formation in the endothelium *in vitro* for the first time. Using a primary cell model to investigate human endothelial functions, we further identified a key role of the ADP-ribosylation factor ARF6 in regulating angiogenic processes and the release of pro-angiogenic factors via a reactive oxygen species (ROS)-dependent pathway.

## Materials and methods

### Reagents

Human umbilical vein endothelial cells (HUVECs), vascular cell basal media, and endothelial cell growth kit BBE were purchased from ATCC (Middlesex, UK). The BCA protein kit was purchased from Thermo Fisher Scientific (Loughborough, UK), while antibodies against ARF6 (58310) and its guanine nucleotide exchange factor (ARF6-GEF) ARNO (10A12) were obtained from Novus Biologicals (Abingdon, UK). Antibodies against β-actin (Ab8227), 2′,7′-dichlorofluorescein diacetate (DCFDA; Ab145286), and assay kits for superoxide dismutase (SOD; Ab65354), catalase (CAT; Ab83464), and glutathione peroxidase (GSH-Px; Ab102530) were purchased from Abcam (Cambridge, UK). NAV2729 and Secin H3 were purchased from Tocris Bioscience (Abingdon, UK); Proteome Profiler Human angiogenesis array and enzyme-linked immunosorbent assay (ELISA) kits for human angiopoietin-2 (DANG20), endothelial growth factor (EGF; DEG00), endoglin (DNDG00), placental growth factor (PIGF; DPG00), prolactin (DY682), and vascular endothelial growth factor (VEGF; DVE00) were all obtained from R&D Systems (Abingdon, UK). One of the most common brands of disposable e-cigarettes sold in the UK was purchased from an online retailer in watermelon flavour in three forms containing 0, 10, and 20 mg of nicotine benzoate equivalent to 0, 1, and 2 mg/mL of nicotine concentration, respectively. All other reagents, including Matrigel^®^ and horseradish peroxidase (HRP)-linked secondary antibodies were purchased from Merck Life Science (Dorset, UK).

### Cell culture and treatments

The HUVECs were cultured in the vascular basal cell media supplemented with endothelial growth kit BBE and 1% penicillin/streptomycin. The cells were used between passages two and seven. E-cigarettes with 50% vegetable glycerine and 50% propylene glycol were vaped as demonstrated previously to produce e-cigarette vapours and were considered to be 100% (Caporale et al., 2019). In brief, the e-cigarettes were vaped using an electric vacuum pump to provide suction at the mouthpiece at the rate of 500 mL/min. This vapour was bubbled through 10 mL of phosphate-buffered saline (PBS) until the canister was depleted, which was denoted by the flashing light on the e-cigarette; thus, 10 mL of the vape fluid is equivalent to one e-cigarette. The e-cigarettes were noted to contain ‘food-grade flavourings’ on the packaging, but the specific chemical nature and concentrations of these are proprietary information. Subsequent dilutions of the vapour were prepared up to 2% and applied to the HUVECs for the durations stated in the legend. A PBS control subject to the same vaping protocol but without any e-cigarette was classified as the eVape vehicle. The HUVECs were exposed to VEGF (100 ng/mL), N-acetyl cysteine (NAC; 1 mM), or H_2_O vehicle or alternatively NAV2729 (5 µM), Secin H3 (10 µM), or DMSO vehicle at the time of eVape treatment (Lizunkova et al., 2019).

### Endothelial cell viability and neovascularisation processes

The viability of the HUVECs was measured following eVape exposure for 24 h and incubation with 3-4,5-dimethylthiazole-2-yl-2,5-diphenyltetrazolium bromide (MTT). The absorbance was then measured at 570 nm using a microplate reader (BMG Labtech) and percentage viability was calculated with normalisation to the 0-µM vehicle. The adhesion and migration of the HUVECs were measured after 6 h of eVape exposure in the presence or absence of VEGF, NAC, NAV2729, or Secin H3 using MTT and scratch assays, respectively (Kertesz et al., 2022). Images of the scratch assay were captured at ×20 magnification using the ZOE™ Cell Imager (Bio-Rad) and quantified into pixels (Barbeau et al., 2013) using MiToBo Analyser software on ImageJ (version 1.52d). The neovascularisation was assessed by plating the HUVECs onto Matrigel^TM^-coated tissue culture ware and treatments for 24 h; these images were again captured at ×20 magnification using the ZOE™ Cell Imager, and the numbers of joints and tubes as well as mesh size in pixels (Barbeau et al., 2013) were measured using Angiogenesis Analyser software on ImageJ.

### Assessment of oxidative stress markers in endothelial cells

The cellular ROS level was assessed using the fluorogenic dye DCFDA. Here, the HUVECs were seeded on black-walled 96-well plates for 24 h, followed by exposure to DCFDA (10 µM) for 30 min at 37 °C in the dark. The DCFDA was rinsed off with prewarmed PBS and replaced with the eVape media or control as well as relevant compound treatments for 2 h. The DCFDA fluorescence was measured at 488/535 nm (excitation/emission) using a fluorescent plate reader (BMG Labtech) and presented in terms of relative fluorescence units.

The cellular GSH-Px, CAT, and SOD activities were quantified using commercially available kits. For all kits, the HUVECs were seeded in 96-well plates and exposed to the eVape media or control as well as relevant compound treatments for 24 h. All assays were performed as per their manufacturers’ protocols, and the absorbances were measured at 340 nm (GSH-Px), 450 nm (SOD), and 570 nm (CAT) using a microplate reader (BMG Labtech). The protein concentration was measured alongside the GSH-Px, CAT, and SOD activities using the protein BCA kit and presented in terms of enzyme units that catalysed the relevant reactions per milligram of protein.

### Protein expression analysis of angiogenic factors and ARF6 signalling

The Human Angiogenesis Proteome Profiler kit (ARY007) was used herein as per manufacturer guidelines. In brief, the HUVECs were exposed to the eVape fluid or relevant treatments prior to collection in a lysis buffer on ice and subsequent centrifugation. The membranes were exposed to the supernatant and antibody mixture overnight at 4 °C. Following three rinses with a wash buffer, the membranes were incubated with streptavidin-HRP for 30 min at room temperature, rinsed again, and visualised with the chemi-reagent mix using the iBright imaging system™ (Invitrogen). Next, densitometry analysis was performed using the Quick Spots image analysis software (R&D Systems). The preloaded templates for ARY007 were aligned with images of the blots before exporting the pixel density and correcting the background density. The percentage change in expression was then calculated from the difference in densities between the vehicle and eVape fluid.

Western blotting was performed next using antibodies specific to human ARF6 and ARNO with β-actin as the reference protein. Here, the HUVECs were exposed to the eVape fluid or relevant treatment prior to lysis with RIPA buffer. The cells were scraped into the buffer over ice, collected, and centrifuged, and the cell pellet was resuspended in Laemmli buffer. The samples (50 µg) were then subjected to immunoblot analysis with 10% SDS-PAGE, 1 μg/mL of the primary antibodies, and 0.2 μg/mL of the secondary antibodies. The membranes were visualised with enhanced chemiluminescence and the iBright™ imaging system; densitometry was performed using the gel analysis software on ImageJ, and the data were normalised with respect to actin.

### mRNA and protein analyses of the angiogenic factors

We analysed the mRNA expression of the angiogenic factors identified by the Proteome Profiler using previously published primers specific to each factor as follows: angiopoietin-2 (forward: GCT​GCT​GGT​TTA​TTA​CTG​AAG​AA; reverse: TCA​GGT​GGA​CTG​GGA​TGT​TTA​G); EGF (forward: CAC​ACC​AAA​CAA​GGA​GGA​GC; reverse: CAT​GAG​AAG​CCC​CAC​GAT​GA); endoglin (forward: CTT​GGC​CTA​CAA​TTC​CAG​CC; reverse: CTT​GAG​GTG​TGT​CTG​GGA​GC); PIGF (forward: GGC​GAT​GAG​AAT​CTG​CAC​TGT; reverse: CAC​CTT​TCC​GGC​TTC​ATC​TTC); prolactin (forward: GCA​GTT​GTT​GTT​GTG​GAT​GAT​T; reverse: GAT​GCC​AGG​TGA​CCC​TTC​GAG​A); VEGF (forward: GCC​TCG​CCT​TGC​TGC​TCT​ACC​TC; reverse: GTT​CTG​TCG​ATG​GTG​ATG​GTG​TG); TATA-binding protein (TBP; forward: CAG​CTT​CGG​AGA​GTT​CTG​GG; reverse: GGG​CAC​TTA​CAG​AAG​GGC​AT); glyceraldehyde 3-phosphate dehydrogenase (GAPDH; forward: TGT​GAG​GAG​GGG​AGA​TTC​AG; reverse: ACC​CAG​AAG​ACT​GTG​GAT​GG) (Kim et al., 2000; Gomez-et al., 2004; Corbacho et al., 2000; Zhou et al., 2014; Yoshimura et al., 2003; Harrington et al., 2018). The total RNA was extracted from the HUVECs using the TRI Reagent® protocol from Merck Life Science before being purified and reverse transcribed as described previously (Aydinlik et al., 2021). The transcripts were measured using polymerase chain reaction (PCR) primers with *TBP* and *GAPDH* as the housekeeping genes. The relative gene expression levels were analysed using the delta-Ct method corresponding to the detected threshold cycles for the target and housekeeping genes.

Next, we performed protein analysis of the angiogenic factors identified by the Proteome Profiler using ELISA kits for each factor (i.e. angiopoietin-2, EGF, endoglin, PIGF, prolactin, and VEGF) in accordance with manufacturer guidelines. In brief, the cell lysates were collected by scraping with RIPA on ice and incubated in plates for 2 h at room temperature. Following rinsing with the wash buffer, the samples were incubated in wells with antibody conjugates for 2 h. The substrate solutions were incubated for 30 min each, and a stop solution was added to visualise the protein expression using a plate reader at 450 nm corrected for optical density at 570 nm.

### Data analyses

All datasets were statistically analysed using GraphPad Prism (version 7.05). For paired groups, the dataset variances were assessed using the Mann–Whitney test followed by t-test. For analyses involving more than two groups, the dataset variances were examined using either one-way or two-way ANOVA with Dunnett’s multiple comparisons post-hoc test where relevant. Statistical significance was considered in cases where the p-value was less than 0.05. All data values are presented as mean ± standard error of the mean (SEM) unless noted otherwise, and the sample size (*n*) is included in each figure legend separately.

## Results

### Nicotine-free eVape exposure significantly increases angiogenic processes in human endothelial cells

**FIGURE 1:**
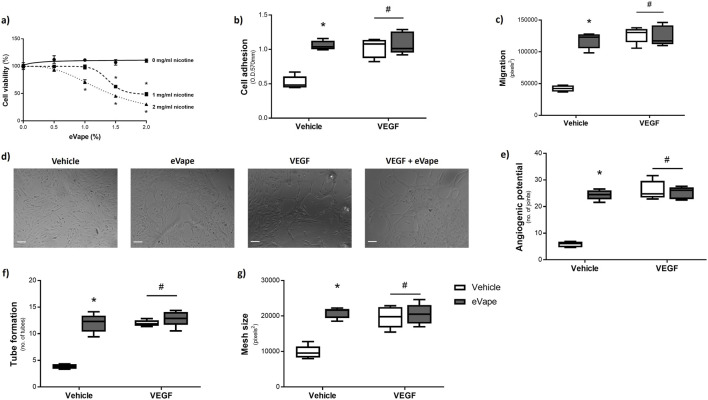
Nicotine-free e-cigarette condensate (eVape) exposure significantly increases angiogenic processes in human endothelial cells. **(a)** Human umbilical vein endothelial cells (HUVECs) were exposed to eVape fluid containing 0, 5, and 10 mg/mL of nicotine for 24 h at concentrations ranging from 0% to 2%. Cell viability was measured using the MTT assay, quantified at 570 nm, and calculated as percentage viability normalised to vehicle. **(b, c)** Endothelial cells were exposed to 2% eVape containing 0 mg/mL of nicotine or vascular endothelial growth factor (VEGF; 100 ng/mL) or vehicle control (H_2_O) for 6 h. **(b)** Cell adhesion was measured using MTT, while **(c)** cell migration was assessed using scratch assay and calculated with MiToBo. **(d–g)** Neovascularisation was measured by culturing cells treated with 2% eVape containing 0 mg/mL of nicotine or VEGF (100 ng/mL) or vehicle control (H_2_O) on Matrigel^TM^-coated wells. Images were captured at ×20 magnification (scale bar: 100 µm; representative images are shown in **(d)**). **(e)** Angiogenic potential, **(f)** tube formation, and **(g)** mesh size were quantified as number of joints, number of tubes, and pixel size, respectively, using Angiogenesis Analyser. The data are presented as mean ± standard error of the mean (SEM), n = 5–6. *p < 0.05 versus vehicle for eVape (0%); ^#^ *p* < 0.05 versus vehicle for VEGF (0 ng/mL).

We have previously demonstrated the negative impacts of eVape on the lung microvasculature, where significant apoptosis and monolayer leakage were noted in response to both nicotine-free and nicotine-containing eVape at concentrations exceeding 2% (Caporale et al., 2019). In the present study, we therefore focused on the effects of eVape fluid concentrations up to 2% with the aim of identifying the impacts on angiogenic processes. We first established the impacts of both nicotine-free and nicotine-containing eVape on the viability of HUVECs. The eVape from e-cigarettes containing 2 mg/mL and 1 mg/mL of nicotine significantly increased cell deaths from concentrations exceeding 1% and 1.5%, respectively (Figure 1a). In contrast, the condensate from nicotine-free e-cigarettes (0 mg/mL of nicotine) had no effect on HUVEC viability up to a concentration of 2% (Figure 1a). Thus, for further experiments, we focused on the use of 2% eVape from nicotine-free e-cigarettes as a non-toxic concentration.

To assess the effects of eVape on angiogenic processes, we used VEGF exposure as a pro-angiogenic factor; here, eVape significantly increased HUVEC adhesion (Figure 1b) and migration (Figure 1c) to similar levels as VEGF exposure alone. Interestingly, co-treatment of HUVECs with both eVape and VEGF did not increase the effects on cell adhesion or migration further (Figures 1b,c). HUVECs plated on Matrigel™ and exposed to eVape demonstrated significant increases in angiogenic potential (Figures 1d,e), tube formation (Figures 1d,f), and mesh size (Figures 1d,g), similar to those seen with VEGF treatment. Likewise, co-treatment with eVape and VEGF did not increase these angiogenic measurements further. These findings demonstrate the significant pro-angiogenic effects of nicotine-free eVape on an *in vitro* model of the vasculature.

### Nicotine-free eVape promotes angiogenic processes by elevating oxidative stress in the endothelium

**FIGURE 2:**
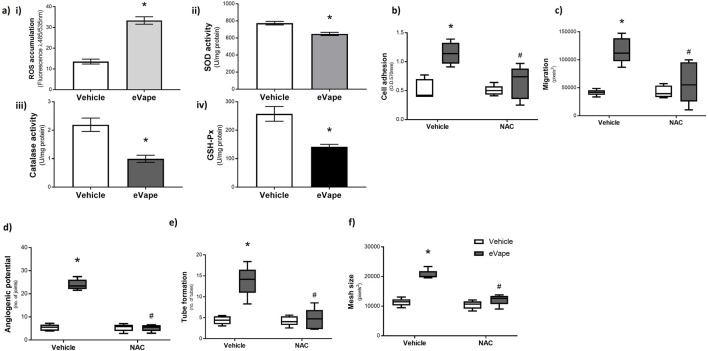
Nicotine-free eVape promotes angiogenic processes by elevating oxidative stress in the endothelium. **(a)** HUVECs were exposed to 2% nicotine-free eVape fluid, and oxidative stress markers were measured: **(i)** reactive oxygen species (ROS) accumulation was assessed as DCFDA incorporation (fluorescence at 485/535 nm) following 2 h treatment; **(ii)** superoxide dismutase (SOD), **(iii)** catalase, and **(iv)** glutathione peroxidase (GSH-Px) activities were measured in units of activity per milligram of protein using colorimetric assay kits at an absorbance of 450 nm. **(b–f)** HUVECs were exposed to 2% nicotine-free eVape fluid in the presence (filled bars) and absence (empty bars) of N-acetyl cysteine (NAC; 1 mM). **(b)** Cell adhesion and **(c)** migration were measured at 6 h of exposure by MTT and scratch assays, respectively, while the angiogenic processes were measured at 24 h of exposure in terms of **(d)** angiogenic potential, **(e)** tube formation, and **(f)** mesh size quantified as number of joints, number of tubes, and pixel size, respectively, using Angiogenesis Analyser. The data are presented as mean ± SEM, n = 5–6. *p < 0.05 versus vehicle for eVape (0%); ^#^ *p* < 0.05 versus vehicle for NAC.

Oxidative stress is a key cellular stress response that has been shown to cause aberrant angiogenesis (Li et al., 2021; Moheimani et al., 2017); previous studies have indicated that eVape fluids act as cellular stressors by increasing oxidative stress (Caporale et al., 2019; Balasubramaniyan et al., 2007). Hence, we sought to understand the roles of nicotine-free eVape exposure on some key markers of oxidative stress in HUVECs (Figure 2a). Endothelial cells exposed to 2% eVape demonstrated a significant increase in ROS accumulation (Figure 2a-i). Given the key roles of the endogenous antioxidant enzymes SOD, CAT, and GSH-Px in clearing excess ROS accumulated in the cells (Ezerina et al., 2018), we investigated the effects of eVape fluid on the activities of these enzymes; eVape fluid exposure resulted in significant decreases in SOD (Figure 2a-ii), CAT (Figure 2a-iii), and GSH-Px (Figure 2a-iv) activities of up to 15.83% ± 4.29%, 51.63% ± 8.52%, and 42.26% ± 7.62%, respectively. To understand whether eVape-induced oxidative stress in the HUVECs was responsible for the increased angiogenic processes observed, we used the pan-antioxidant NAC (Yao et al., 2024; Ikeda et al., 2005). Co-treatment of the HUVECs with NAC and eVape resulted in significant decreases in endothelial cell adhesion (Figure 2b) and migration (Figure 2c) compared to eVape treatment alone. Similarly, NAC blocked eVape-induced angiogenic processes such that the number of joints (Figure 2d), amount of tube formation (Figure 2e), and mesh size (Figure 2f) returned to baseline levels. These findings show that nicotine-free eVape exacerbates oxidative stress in the endothelium to increase neovascularisation *in vitro*.

### Nicotine-free eVape exposure significantly upregulates angiopoietin-2, endoglin, PIGF, and VEGF but significantly downregulates EGF and prolactin in endothelial cells

**FIGURE 3:**
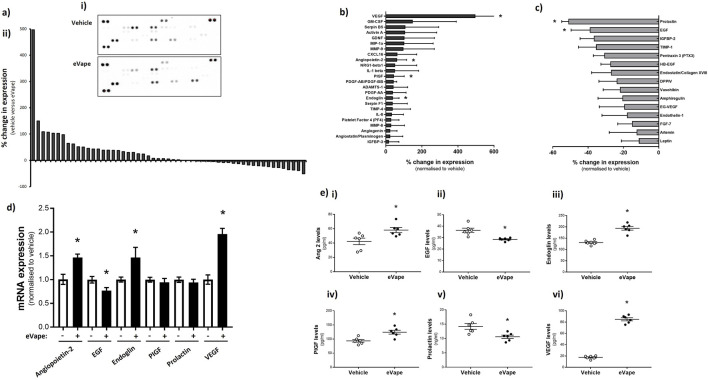
Nicotine-free eVape exposure causes significant upregulation of angiopoietin-2, endoglin, placental growth factor (PIGF), and VEGF as well as significant downregulation of endothelial growth factor (EGF) and prolactin in endothelial cells. HUVECs were exposed to 2% nicotine-free eVape fluid for 24 h. **(a–c)** Cell lysates were used for the Proteome Profiler Angiogenesis array membranes. Changes in the expression of all angiogenesis-related proteins in array **(a) (ii)** were quantified using the membranes (representative image in **(a) (i)**). Specific analyses of the **(b)** upregulation and **(c)** downregulation of angiogenesis-related proteins on the membranes were quantified. **(d)** mRNA expression of angiopoietin-2, EGF, endoglin, PIGF, prolactin, and VEGF were measured by RT-PCR using specific primers. **(e)** Protein expression of **(i)** angiopoietin-2, **(ii)** EGF, **(iii)** endoglin, **(iv)** PIGF, **(v)** prolactin, and **(vi)** VEGF were measured using specific ELISA arrays. The data are presented as mean ± SEM, n = 5–6. *p < 0.05 versus vehicle for eVape (0%).

Given the range of signalling pathways in endothelial cells that regulate angiogenic processes, we used a protein array to identify the molecular mechanisms by which eVape regulated new blood vessel formation. From the HUVEC lysate, we identified 24 proteins with increased expression by more than 10% following eVape exposure compared to the vehicle (Figure 3a). Of these proteins, four were significantly upregulated, namely, VEGF, angiopoietin-2, PIGF, and endoglin (CD105) (Figure 3b). In contrast, 15 proteins were showed reduced expression by more than 10% following eVape exposure, of which two were significantly downregulated, namely, prolactin and EGF (Figures 3a,c). We next confirmed the eVape-induced changes in expression at both the mRNA and absolute protein levels using reverse-transcription quantitative PCR and ELISA, respectively. The mRNA expression of angiopoietin-2, endoglin, and VEGF were significantly upregulated with eVape exposure, while the EGF mRNA level was significantly reduced; however, the mRNA expression of PIGF and prolactin were not affected by eVape exposure (Figure 3d). In contrast, the protein expression measured by ELISA (Figure 3e) mirrored the significant changes in expression noted with the protein arrays for angiopoietin-2, EGF, endoglin, PIGF, prolactin, and VEGF (Figures 3a–c). Notably, we observed the most dramatic change in expression for VEGF with eVape exposure, which resulted in a 47.55% ± 7.01% increase in mRNA expression and 79.44% ± 1.19% increase in protein expression (Figures 3d,e). These studies indicate that the pro-angiogenic signalling molecules stimulated by nicotine-free eVape exposure in human endothelial cells are VEGF, angiopoietin-2, PIGF, and endoglin (CD105). The studies further demonstrate that downregulation of prolactin and EGF may play roles in nicotine-free-eVape-induced angiogenesis; however, eVape exposure does not appear to exert transcriptional regulation on PIGF or prolactin.

### ARF6 activation by ARNO induced by nicotine-free eVape regulates angiogenic processes in endothelial cells

**FIGURE 4:**
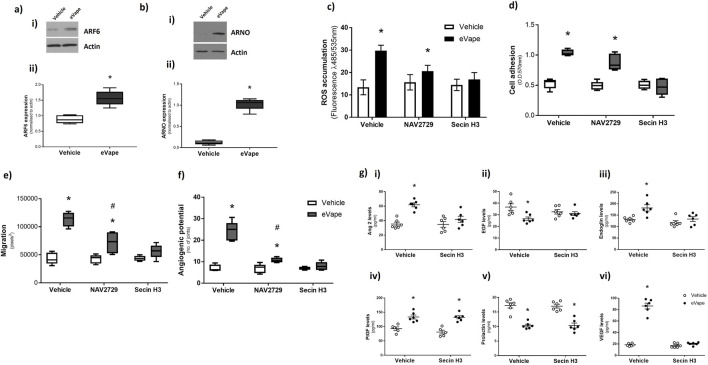
Activation of ARF6 by its guanine nucleotide exchange factor (ARNO) regulates angiogenic processes in endothelial cells induced by nicotine-free eVape. **(a, b)** HUVECs were exposed to 2% nicotine-free eVape fluid for 24 h, and the cell lysates were analysed by Western blotting for **(a)** ARF6 or **(b)** ARNO expression. The Western blotting membranes (representative blots are shown in **(i)**) were quantified and normalised to the actin expression shown in **(ii)**. **(c–f)** HUVECs were treated with ARF6 inhibitor (NAV2729; 5 µM), ARNO inhibitor (Secin H3; 10 µM), or dimethyl sulfoxide (DMSO) as vehicle control at the same time as eVape exposure. **(c)** ROS accumulation was assessed by DCFDA incorporation (fluorescence at 485/535 nm) following 2 h of treatment. **(d)** Cell adhesion, **(e)** migration, and **(f)** angiogenic potential were measured following **(d, e)** 6 h and **(f)** 24 h of treatments. **(g)** Protein expression of **(i)** angiopoietin-2, **(ii)** EGF, **(iii)** endoglin, **(iv)** PIGF, **(v)** prolactin, and **(vi)** VEGF were measured using specific ELISA arrays following 24 h of treatment with 2% nicotine-free eVape fluid in the presence (closed circles) and absence (open circles) of Secin H3 (10 µM). The data are presented as mean ± SEM, n = 5–6. *p < 0.05 versus vehicle for eVape (0%); #*p* < 0.05 versus vehicle for NAV2729 and Secin H3 (DMSO).

Our previous studies have demonstrated a key role of ARF6 in regulating eVape-induced pulmonary endothelial cell function (Caporale et al., 2019). Given the striking increases in VEGF mRNA and protein expression observed in the HUVECs following eVape exposure (Figure 3) as well as the close association between ARF6 and VEGF signalling (Flaxel et al., 2020), we examined the link between ARF6 and eVape-induced angiogenesis in HUVECs. We observed significant increases in the protein expression of both ARF6 (Figures 4a-i,a-ii) and ARNO (Figures 4b-i,b-ii) upon exposure to nicotine-free eVape. Therefore, we assessed whether the selective ARF6 inhibitor (NAV2729) or cytohesin inhibitor (Secin H3) had any effect on eVape-induced oxidative stress or angiogenic processes. The HUVECs exposed to both NAV2729 and eVape demonstrated no significant changes in ROS accumulation (Figure 4c) or cell adhesion (Figure 4d). Interestingly, although eVape-induced endothelial cell migration (Figure 4e) and angiogenic potential (Figure 4f) were significantly reduced with NAV2729 treatment, these did not return to the baseline levels. In contrast, co-treatment with Secin H3 completely attenuated eVape-induced ROS accumulation (Figure 4c), cell adhesion (Figure 4d) and migration (Figure 4e), and angiogenic potential (Figure 4f). Likewise, inhibition of ARNO through Secin H3 abrogated eVape-induced overexpression of angiopoietin-2 (Figure 4g-i), endoglin (CD105) (Figure 4g-iii), and VEGF (Figure 4g-vi) as well as eVape-induced downregulation of EGF (Figure 4g-ii). However, Secin H3 co-treatment with eVape did not impact PIGF (Figure 4g-iv) and prolactin (Figure 4g-v) expression levels. These findings confirm the key role of ARF6 in eVape-induced endothelial injury while demonstrating that inhibition of ARNO significantly attenuates eVape-induced oxidative stress and angiogenic processes *in vitro*.

## Discussion

There is growing evidence that although e-cigarettes are a recommended alternative to cigarette smoking, their use may be associated with significant negative health impacts. In the present study, we identified the effects of nicotine-free e-cigarette condensates on endothelial cell functions and increased angiogenic processes for the first time. Our findings demonstrate that nicotine-free eVape exerts significant stress on the endothelial cells through elevated ROS accumulation and downregulated activities of several endogenous antioxidant enzymes to increase endothelial cell adhesion, migration, and tube formation. We further discovered that eVape-induced angiogenesis *in vitro* is mediated by ARF6 regulation through ARNO. These findings provide a possible molecular mechanism underlying eVape-induced vascular dysregulation and demonstrate that nicotine-free e-cigarettes could be potential stressors to the endothelium.

The physiological need for angiogenesis ranges from development through wound healing; however, upregulated or dysregulated angiogenesis in adults contributes to various diseases or pathological states. For example, visual impairment in patients with neovascular age-related macular degeneration is a result of excessive overgrowth of the choroidal vessels, leading to leakage and haemorrhage (Abumiya et al., 1999). During ischaemia, there is restricted oxygen supply to the cardiovascular system that results in tissue starvation and induction of the hypoxia-induced transcription factor (HIF1-α) to drive VEGFA transcription and act as a pro-angiogenic switch (Shweiki et al., 1992; Baumgartner et al., 1998). This response triggers angiogenesis that results in collateral vessel development to re-oxygenate the tissues (Clayton et al., 2008; Carmeliet, 2005). In solid tumours, chronically hyperactivated angiogenesis is initiated as a hallmark of cancer through activation of the pro-angiogenic switch largely by upregulation of *VEGF* gene expression as a result of both oncogene signalling and response to the hypoxia seen in the cores of solid tumours (Bielenberg and Zetter, 2015). This hyperactivated angiogenesis results in dysfunctional and hyperpermeable vasculature that sustain tumour growth and support the formation of metastasis (Hanahan, 2022; Ollauri-Ibanez et al., 2020; Pasut et al., 2021). While there are significant heterogeneities in the endothelial cells and different modes of angiogenesis, the processes involved in new vessel formation are well-understood and consistent across different vascular beds with elevated pro-angiogenic signals, such as VEGF, as well as increased cellular migration, differentiation, and adhesion (Leung et al., 1989; Gerhardt et al., 2003; Nejabati et al., 2017). In the present study, we investigated the effects of e-cigarette condensates on these endothelial cell processes to understand the pro-angiogenic effects of such cell stressors. In addition to significant increases in angiogenic processes, we observed that endothelial cell migration, adhesion, and new vessel formation were similar to those seen with VEGF exposure. VEGF is a widely expressed angiogenic factor that promotes new vessel formation by regulating filopodia tip cell formation (Nejabati et al., 2017). Interestingly, we noted that nicotine-free eVape also significantly increased VEGF mRNA and protein expression in the endothelium *in vitro.* It is therefore likely that eVape induces a pro-angiogenic signalling switch in the endothelial cells. Indeed, although VEGF protein is most highly expressed following eVape exposure, our study demonstrates that other pro-angiogenic signals are upregulated in these settings along with increased angiopoietin-2, PIGF, and endoglin (CD105) expression. Both PIGF and endoglin act as accessories to promote the VEGF signalling cascade; PIGF directs VEGF to its receptor (VEGFR2) to promote angiogenesis, whereas endoglin acts as a scaffold to hold NRP1 and VEGFR2 together to promote VEGF-induced angiogenesis (Sharma et al., 2024; Maisonpierre et al., 1997). In the past, angiopoietin-2 has been shown to act as either a pro- or anti-angiogenic signal depending on the tissue and stimulus (Asahara et al., 1998; Lobov et al., 2002); however, it is now understood that in the presence of VEGF, angiopoietin-2 induces endothelial cell migration and sprouting to promote angiogenesis (Mehta and Besner, 2007). As exposure to nicotine-free eVape significantly increased VEGF mRNA and protein expression in the endothelial cells, it is likely that angiopoietin-2 acts as a pro-angiogenic signal in these events. In contrast, we observed decreases in EGF and prolactin expression following treatment with eVape. Prolactin has been shown to exert an anti-angiogenic switch by stimulating plasminogen activator inhibitor type 1; however, EGF is a significant pro-angiogenic stimulant (Nezu et al., 1992; Struman et al., 1999; McVicar et al., 2007). Therefore, eVape appears to promote angiogenesis through three mechanisms as follows: 1) by modulating the cell process itself, that is, via increased endothelial cell adhesion, migration, and tube formation; 2) by stimulating the release of pro-angiogenic signalling as a positive feedback to maintain the angiogenic processes; 3) by inhibiting the anti-angiogenic prolactin signal to remove negative regulation of new blood vessel formation. EGF exerts a pro-angiogenic effect through upregulated VEGF secretion; therefore, it is possible that the reduced EGF expression in response to eVape exposure is a form of negative feedback as the endothelium attempts to control the exacerbated VEGF levels (Crotty Alexander et al., 2018). This could result in a combined and continuous pro-angiogenic switch in individuals who use e-cigarettes, which could mirror the pathological angiogenesis seen in ischaemia or cancer. However, further studies are needed to investigate the pathologies resulting from such long-term pro-angiogenic signalling in individuals who use nicotine-free e-cigarettes.

While e-cigarettes are still in their early stages of use to allow good understanding of the long-term health impacts, there is evidence in literature that eVape exposure can regulate angiogenesis. *In vivo* mouse models of e-cigarette exposure show increased new vessel formation in cardiac tissues and elevated pro-angiogenic markers, such as angiopoietin, whereas *in vivo* rat models have demonstrated decreased micro-vessel density and reduced VEGF expression (Liu et al., 2022; Cucina et al., 2000a; Cucina et al., 2000b). *In vitro* studies have demonstrated increased ROS accumulation and angiogenesis markers along with impaired responses to VEGF (Jaleel et al., 2021; Shi et al., 2019). However, to date, all these studies were conducted on e-cigarettes containing nicotine. In the present study, we focused our attention on nicotine-free e-cigarettes as it is well-established that nicotine is a potent stimulus of angiogenesis via direct actions on the nicotinic receptors (Mannell et al., 2012). These nicotine-dependent calcium channels stimulate eNOS expression and promote the release of bFGF, prostacyclin, and endothelin from the endothelial and smooth muscle cells to increase micro- and macro-vascular injury (Zhang et al., 2001b; Matheson et al., 2024). Research with human participants show that e-cigarette use causes impaired micro- and macro-vascular functions along with excessive shedding of the extracellular vesicles from the endothelium (Mobarrez et al., 2020; Kelesidis et al., 2021). However, in these studies, there is no significant association between vascular damage and the nicotine content of e-cigarettes, suggesting that there are components within e-cigarettes other than nicotine that can cause vascular injury. In agreement with this suggestion, we previously showed that nicotine-free eVape significantly increased endothelial barrier disruption in the pulmonary endothelium (Caporale et al., 2019). Similar to the present study, eVape-induced endothelial disruption was associated with excessive ROS accumulation. These findings suggest that eVape is a pro-oxidant cell stressor, and these effects have been observed in immune cells from human participants using e-cigarettes (Kinnula, 2005). In the present study, we noted that use of a pan-antioxidant like NAC significantly attenuated vascular injury and inflammation *in vitro*; however, studies have shown that exogenous antioxidant supplementation is not sufficient to protect against cigarette-smoke-induced inflammation (Kosmider et al., 2014). Therefore, there is a need to further consider methods to tackle the pro-oxidant responses seen individuals using e-cigarettes.

In the present study, we used a common brand of e-cigarette containing a 50:50 ratio of vegetable glycerine and propylene glycol. While there may also be proprietary natural and artificial flavourings present in the e-cigarette fluid that supply the watermelon flavour, vegetable glycerine and propylene glycol are the major sources of toxicants, such as formaldehyde, acetaldehyde, and acrolein, generated from e-cigarette use (Wang et al., 2017; Tyihak et al., 2001). It is therefore likely that these compounds are the major causes for the increased oxidative stress and angiogenic processes in the endothelial cells noted herein. Indeed, formaldehyde and acetaldehyde have been found to increase endothelial cell proliferation, consistent with a neovascularisation phenotype (Wohlfart et al., 2022; Wheat et al., 2011). Acrolein has been shown to have an anti-angiogenic effect linked to reduced VEGF mobilisation as well as elevate the VEGF level in a model of reactive airway dysfunction (Kim et al., 2019; Pinto et al., 2022). Therefore, it is likely that the precise chemical composition of the e-cigarette fluid and the specific breakdown constituents from heating the fluid to form eVape determine the specific biological effects of the e-cigarette. Furthermore, some of the unknown chemicals that have been identified in some brands of e-cigarettes, such as metals, nitrosamines, and polycyclic aromatic hydrocarbons, as well as flavouring molecules like isoamyl acetate could be working in synergy to cause the oxidative and pro-angiogenic effects on endothelial cells (Shehata et al., 2023; Herringt et al., 2015). Further studies are needed to establish a thorough chemical analysis of eVape to demonstrate the concentration ranges for all constituents and investigate how each constituent impacts the angiogenic processes. However, the dramatic differences in e-cigarette fluid constitution between different brands must be noted (Spindle et al., 2018), which pose significant challenges with regard to the universality of our findings across different commercially available e-cigarettes. Furthermore, there are vast differences in the frequency of e-cigarette use by individuals and the amounts that they vape during any given period. For example, despite standardising to 10 puffs per smoke, one study showed that an average e-cigarette puff volume was 96.8–133.9 mL, whereas another study showed a range of 331.2–519.6 mL (Hiler et al., 2020; Daher et al., 2010). In the present study, we focused on the impacts of 2% eVape exposure for 24 h on the angiogenic processes given our previous findings that this was a sub-lethal dose. While it is difficult to correlate this concentration with e-cigarette use, given that one e-cigarette is equivalent to 100% of the dose in our study, our findings suggest that even short-term exposure to an e-cigarette of 2% concentration could have negative impacts on blood vessel functions. Further studies are thus needed to investigate how these findings translate to those who regularly use e-cigarettes and to better understand the vascular functions in these individuals.

There are a limited number of studies that have investigated the molecular mechanisms underlying vascular injuries observed in individuals using nicotine-free e-cigarettes. In a screening procedure for adherens-junctions-related genes, we recently demonstrated overexpression of the ADP-ribosylation factor 6 in lung microvascular endothelial cells following exposure to nicotine-free eVape (Caporale et al., 2019). In the present study, we similarly identified that nicotine-free eVape increased ARF6 expression in HUVECs that was accompanied by increased levels of the ARF6-GEF protein ARNO. ARNO has specificity for both ARF1 and ARF6, and ARF1 has been shown to modulate VEGF-induced signalling in the endothelium (Frank et al., 1998). However, ARNO has sixfold higher binding affinity to ARF6; therefore, it is likely that ARNO upregulation following exposure to nicotine-free eVape could stimulate ARF6 activity by catalysing ARF6-GDP to ARF6-GTP (Santy et al., 2001; Francis et al., 2023). ARF6 has been identified to localise to filamentous actin to maintain endothelial cell polarity and controlled angiogenic sprouting, whereas ARNO maintains VEGFR2 expression to support VEGF-induced neovascularisation (Lizunkova et al., 2019; Rosenberg et al., 2023). We observed that chemical inhibition of ARNO (Secin H3) but not ARF6 (NAV2729) exhibited significant and robust abrogation of eVape-induced angiogenesis and pro-angiogenic signalling. It is worth noting that NAV2729 acts by inhibiting the ARF-GEFs Brag2 and ARNO rather than directly binding to ARF6 (Chen et al., 2014). Further, NAV2729 binds directly to key members of the Rho GTPase pathway that is closely aligned with pro-angiogenic processes (Chen et al., 2014). Therefore, it is likely that molecular inhibition of ARF6 through siRNA would be more precise at establishing the role of ARF6 on eVape-induced angiogenesis. Given the role of ARNO in promoting VEGF-dependent Akt phosphorylation and VEGFR2 expression, it is possible that nicotine-free eVape stimulates ARNO to promote angiogenesis by activating ARF6 and by upregulating VEGFR2 to increase the ability of the endothelial cells to respond to the elevated VEGF release observed (Lizunkova et al., 2019). Hence, further studies are needed to understand the components of eVape that cause the responses observed herein and their upstream mechanisms; there is also a need to establish how nicotine-free eVape as a cellular stressor regulates ARNO expression. Further studies are also required to understand the longer-term effects of exposure to nicotine-free eVape as well as the effects of withdrawing exposure to eVape.

## Data Availability

The original contributions presented in the study are included in the article/Supplementary Material, further inquiries can be directed to the corresponding author/s.
